# Mexicans vs Central Americans: Violented Migrants Crossing Mexico

**DOI:** 10.1007/s40615-023-01767-3

**Published:** 2023-08-30

**Authors:** Miguel Á. Fernández-Ortega, René Cerritos-Flores, Omar Rodríguez-Mendoza, Rocío Dávila-Mendoza, Brandon Salas-Sánchez, Daniel A. Muñiz-Salinas, Yuriana Martinez-Orea, Investigation team, Yossadara Luna Téllez, Andrea Macías Silva, Flor Mariana Sanchez Nuñez

**Affiliations:** 1 Centro de Investigación en Políticas, Población y Salud (CIPPS), Universidad Nacional Autónoma de México (UNAM), Mexico City, Mexico; 2 Subdivisión de Medicina Familiar, Facultad de Medicina, Universidad Nacional Autónoma de México (UNAM), Mexico City, Mexico; 3 Department of Ecology and Natural Resources, Facultad de Ciencias, Universidad Nacional Autónoma de México (UNAM), Mexico City, Mexico

**Keywords:** Sexual violence, International migration, Violence, Mexican border, Tijuana

## Abstract

**Introduction:**

The World Health Organization considers that migrants who pass through the Mexico–US walkway suffer high levels of violence, compared to other regions of the world, mainly women. This study aims to identify the factors associated with the types of violence suffered by migrants in transit through Mexico to the USA.

**Design:**

A cross-sectional, exploratory, retrospective, and observational study was conducted. A questionnaire of 46 variables was applied, divided into four sections: sociodemographic background, leaving the home, transit, and stay at the border. Questions about different types of direct violence were included. The survey was applied to 612 Mexican and Central American migrants who were in the Chaparral customs office and in five shelters in Tijuana City, on the U.S.–Mexico border. The results were analyzed using descriptive techniques and multivariate analysis of main and inferential components, using the statistical program R.

**Results:**

The higher vulnerability of Central American migrants compared to Mexicans was documented, specially of women that proportionally were the most negatively affected victims including all types of violence, making it evident that one of each four was violented sexually and among them, only 50% asked for medical assistance. The multivariate analysis determined that the duration of the trip, and the type of transport can generate greater violence.

**Conclusions:**

The results highlight the greater vulnerability of Central American migrants in their transit through Mexico, mainly women and, likewise, the lack of effective public policies that guarantee the protection of the health, safety, and human rights of migrants.

## Introduction

World Health Organization (WHO) defines violence as “The intentional use of physical force or power, consummated or as a threat, against oneself, another person, a group of people or community, resulting in or a high probability of causing injury, death, psychological harm, developmental disorders or deprivation” [1].

The governments of Mexico and the USA have strengthened immigration laws over the past 6 years, increasing the number of apprehensions of migrants passing through Mexico, from 8500 in January 2019 to 13,500 in the same month of 2020 [2]. This has led irregular migrants to seek for new routes to avoid immigration checkpoints that are less traveled, but may be more dangerous [2–6].

It is important to consider that the Mexico–US corridor constitutes the main migratory path in the world, representing 5.3% of global migration in 2020 [2]. In this mass migration, both Mexican and other Latin American migrants have suffered violence in all its forms [2, 3, 7–9].

The Missing Migrants project of the International Organization for Migration (IOM), reported that between 2014 and 2018, at least 3000 people lost their lives while crossing the American continent and of these, more than 60% died on the border between Mexico and the USA [2]. The lack of official records on irregular migrants hinders their “visibility” during transit through Mexico, which means that a large number of crimes go unpunished [7].

Gender-based violence during migration flows can be aggravated by the vulnerability of women [10–12]. According to Amnesty International, one in six women suffer sexual violence during their journey to the USA; it has even been reported that Central American women who cross through Mexico take prophylactic contraceptives to avoid becoming pregnant after a sexual attack [13, 14].

This study aims to identify factors that may be associated with the types of violence suffered by migrant women transiting through Mexico to the USA and the use of health services.

## Materials and Methods

A cross-sectional, exploratory, retrospective, and observational study was conducted. We applied as an instrument a questionnaire of 46 variables, divided into four sections: sociodemographic background, leaving the home, transit, and staying at the border. Questions about different types of direct violence were included. Prior to the application of the instrument, a pilot test was developed electronically in a group of 20 migrants with characteristics similar to those of the final sample, and semantic corrections were made to the instrument. The validation of appearance and content was analyzed and approved by eight experienced academics from the Faculty of Medicine of the Universidad Nacional Autónoma de México (UNAM).

The survey was applied in print to 612 Mexican and Central American migrants who were in the Chaparral Garita (customs) and in five shelters in the city of Tijuana, on the border of Mexico and the USA: Casa de los Pobres A.C., Casa del Migrante, Salesian breakfast bar “Padre Chava”, Instituto Madre Asunta A.C., and Ejército de Salvación A.C.

Respondents filled out the informed consent form and signed it with the support of a medical interviewer. They were guaranteed that their participation would be anonymous, voluntary, and without repercussions of any kind. The results were captured in Excel and then the statistical analysis was performed using descriptive and inferential techniques through the statistical program R.

The project was registered and approved by the Research Commission and the Ethics Committee of the Faculty of Medicine of the Universidad Nacional Autónoma de México: FM/DI/113/2019.

## Results

The application of the surveys was carried out between November 2019 and March 2020. For the purposes of this study, only questions related to the transit phase were considered. Of this universe of 612 migrants, 538 (87.90%) were Mexicans and 74 (12.10%) were Central Americans, mainly from Guatemala and Nicaragua. Of these, 490 were Mexican males (91%) and 48 were (8.9%) Mexican females, while 52 (70.27%) were Central American males and 22 (29.72%) were Central American females.

**Fig. 1:**
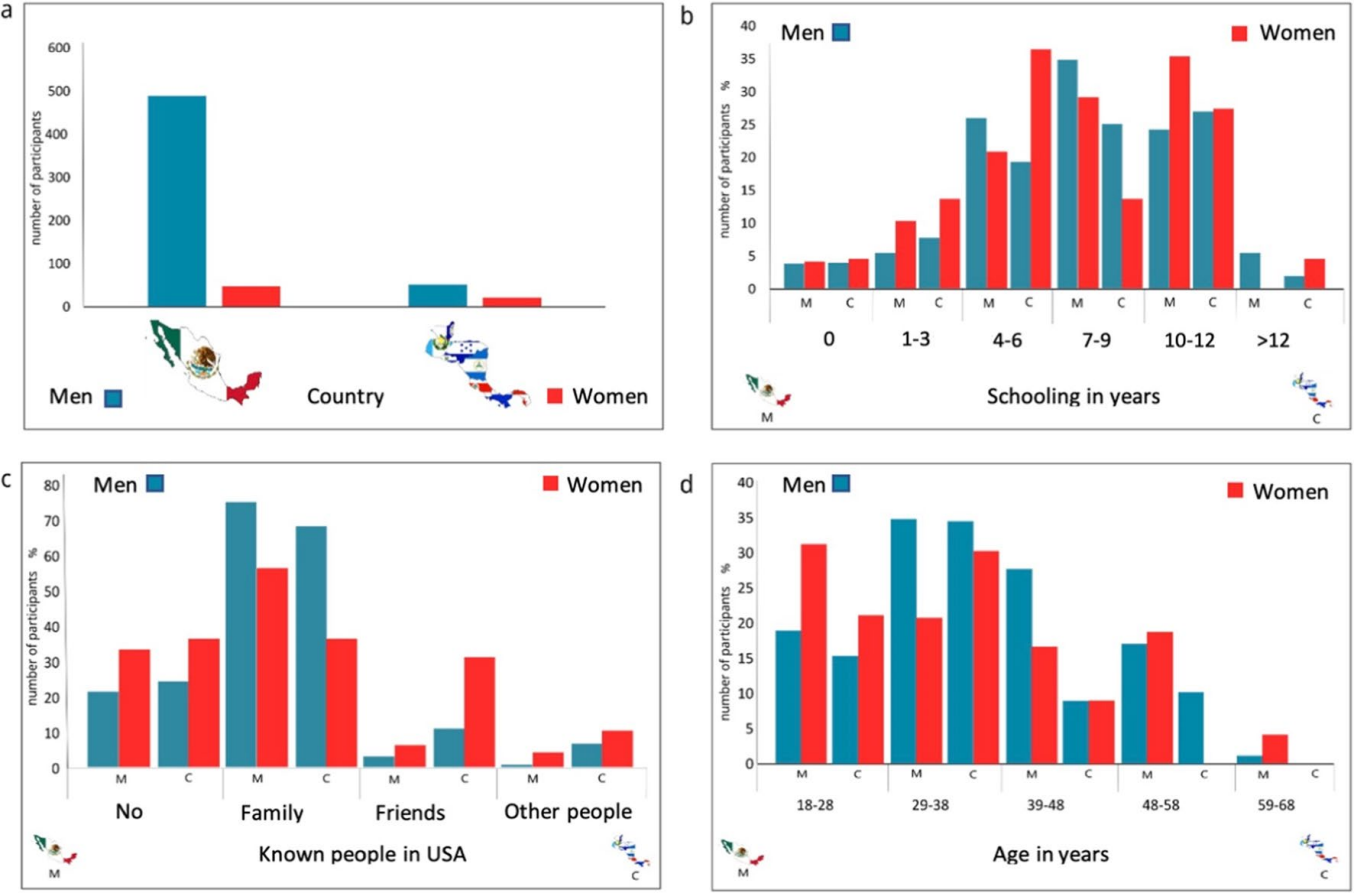
Characterization of violence by nationality, sex, schooling, and family/friends in the USA: Mexicans vs Central Americans. Source: own elaboration. Data on **a** nationality are observed. **b** Age. **c** Schooling in years. **d** Family or friends in the USA

Our study highlights that Mexicans, regardless of gender, have in proportion a greater number of relatives or friends in the USA (78%), in relation to Central American migrants (68%). The schooling of Central American and Mexican migrants did not show significant differences, with the average schooling for the four categories (Mexican females, Mexican males, Central American females, and Central American males) being 8 years. Regarding age, we found out that Central American women are younger than Mexican women, finding in general two age ranges: 18–28 and 29–38 years, with proportions of 0.49 and 0.51 respectively, while for Mexican women, they were distributed in all ages, in fact there were women of 49–58 and 59–68 years, with proportions of 0.13 and 0.09, respectively. With respect to the reasons for emigrating, it stands out that for Mexican women they were mainly economic (78%) and reunification with relatives (16%), while for Central American women it was social violence (72%), followed by economic, family reunification, and political persecution with 9% each (see Fig. 1).

The types of violence during the transit phase included in this work are classified as: physical, psychological, robbery, kidnapping, and sexual type. A differentiation was made between Mexican and Central American migrants.

**Fig. 2:**
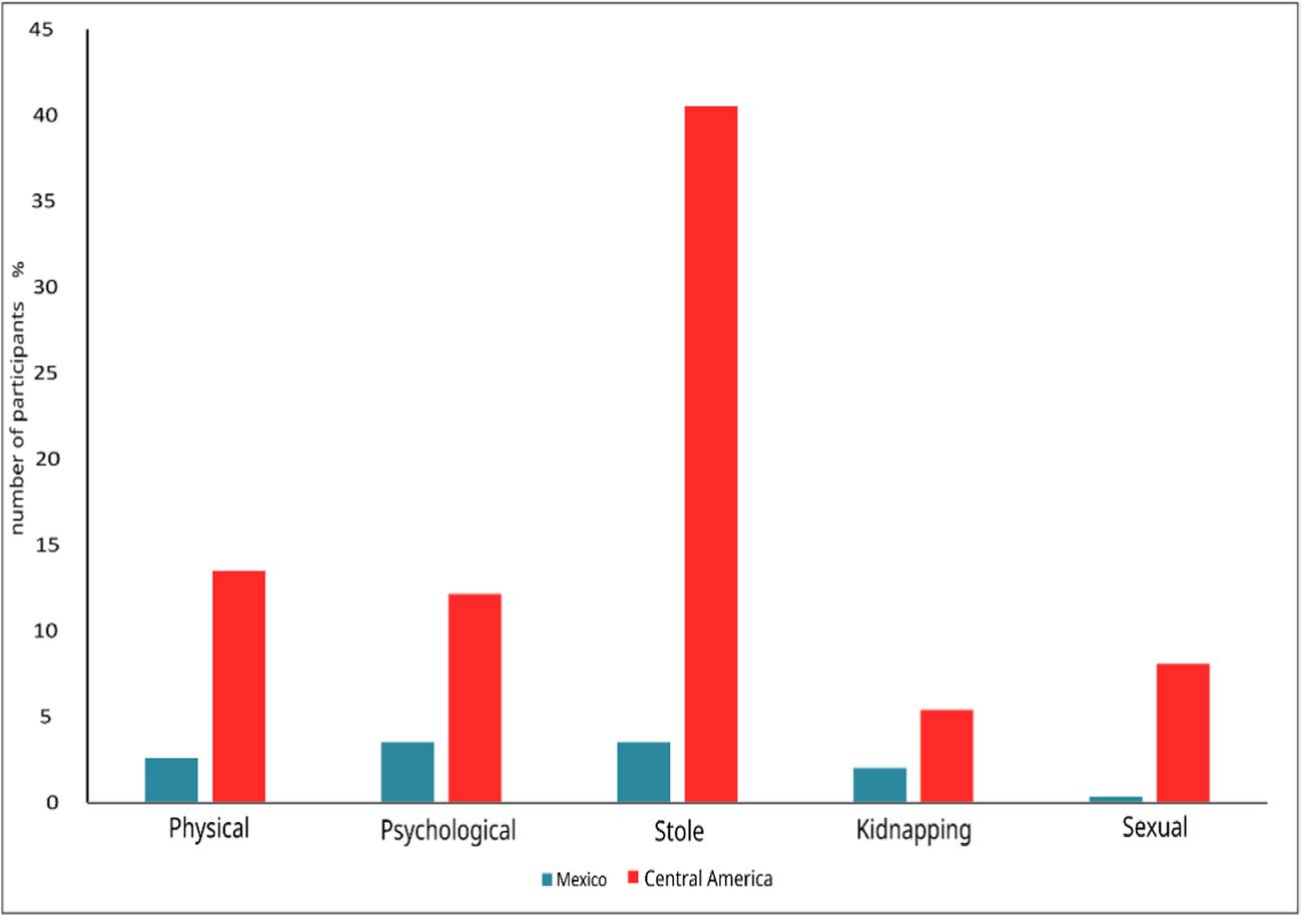
Proportion of violent acts towards Mexican and Central American migrants. Source: own elaboration. It is observed that robbery in Central American migrants is the largest violence act suffered during their transit phase

We observed that robbery is the most frequent violent act with 41% for Central Americans and 4% for migrants from Mexico. On the other hand, kidnapping was the least frequent violent act, being also higher for Central Americans than for Mexicans (1.5 and 6%, respectively). There were significant differences in the “type of aggression” suffered between the two groups of Mexican and Central American migrants. The Y axis represents the proportion of people in a total population; for example, in Fig. 2, the physically violated migrants were 14 and 10 for Mexico and Central America, respectively; however, although these values seem very similar, a proportionality ratio was determined in this case of a total of 538 and 74 migrants, respectively, for Mexico and Central America.

In all cases, violent behaviors were more frequent towards Central American migrants than towards Mexicans, even by an order of magnitude. With respect to sexual violence, the frequency was greater than an order of magnitude for these two groups, although, for Mexican migrants, out of every 1000 people, 3 were sexually assaulted, while for Central Americans the value was 80. The *X*^2^ test indicates that the null hypothesis is rejected by finding significant differences between the defined categories, by obtaining a value of 6.39 (*df* = 1, critical value = 3.84, *p* = 0.05).

**Fig. 3:**
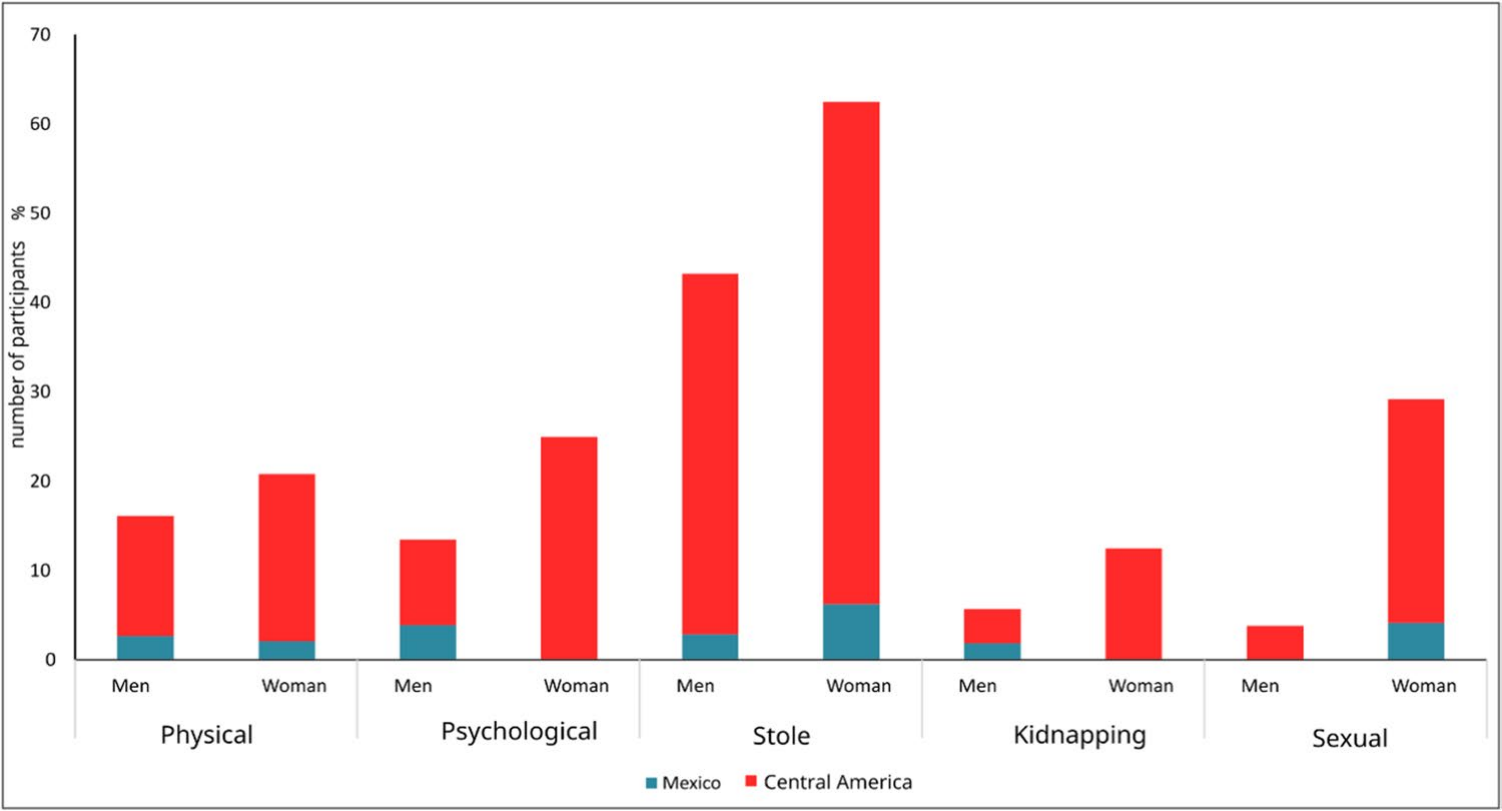
Proportion of violence suffered by gender in Mexican and Central American migrants. Source: own elaboration. Central American women are more vulnerable to all violence types compared to men in both studied regions and even when compared to mexican women

The proportion of violent acts was also analyzed by gender, dividing the total for women and men. From this, the proportions in the five types of violence were calculated. Central American women were found to be at least one order of magnitude more vulnerable than Mexican women and much higher when compared to men from both regions. The interviewees mentioned that theft is the most frequent criminal activity, suffered especially by Central American migrants: 39.5% men and 60.5% women, the latter being more susceptible in almost 20%. With regard to sexual violence, the proportions indicate that one in four Central American women were subjets to this type of violence, and the majority raped. Furthermore, all these women are not only sexually violated but also suffered robbery, kidnapping, and physical and psychological violence. Figure 3 shows in blue and red the proportions of people who were violated in the different areas, and in general, in most cases, the difference is almost an order of magnitude higher in Central American women.

The PCA analysis showed that the environment that exists during the transit phase, before a woman is violated, can depend on several factors: the duration of the trip, if the person was accompanied, and the means of transport used. A total of 90% reported the bus as the main means of transportation; however, in most cases, the journey was combined, including train and/or walking. It was observed that Central American women took up to 109 days on average to reach the border of the USA from their home, in contrast to Mexican women who took an average of 3.7 days. It is noteworthy that Central American migrants who traveled accompanied by their children took fewer days on their trip, with an average of 74.4 days. Regarding the use of medical services, both Central Americans and Mexicans reported having received medical help in 50% of cases, even after a sexual violation, both by public and private institutions or by non-governmental organizations.

In a principal components analysis, the relationships between the variables can be seen reflected in a Cartesian plane where *X* represents a first component and *Y* represents the second component. Each of the variables is represented by a vector, which has a direction within the *X*,*Y* plane. When two vectors take opposite directions, it indicates that the relationship between those variables are inversely proportional. Likewise, each of the sampling units, that is, the surveyed individuals, will be positioned in a space within the *X*, *Y* plane, in such a way that the proximity between them will indicate a similarity product of the analyzed variables.

**Fig. 4:**
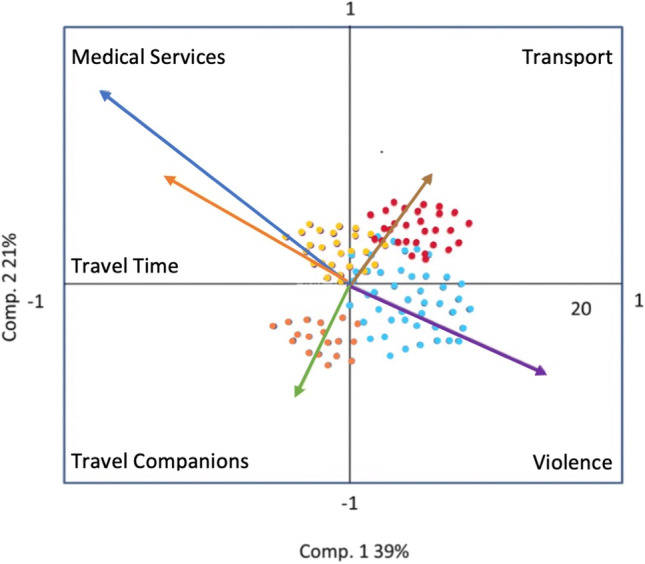
Principal component analysis of violence factors. Source: own elaboration. Principal component analysis where the relation between variables is observed (arrows). Each color dot represents an individual in the survey: closeness within the Cartesian plane indicates similarity in the analyzed variables. Orange (Mexican Men HMx), red (Mexican Women MMx), blue (Central America Men HCA), yellow (Central America Women MCA)

Figure 4 indicates the direction between the variable “violence” and the variable “medical services” is inverse, which indicates that while the violence frequency is higher, the lower is the search for medical attention. An inverse relation was observed between the variables “violence” and “travel time” which is interpreted as the highest value in the relation to “violencia” which was “there was no violence“ (value = 8). Specifically for central american women, sexual violence is closely related to travel time. For example, the mean value of travel time for central american women to cross Mexico was 109 días, while for mexican women was 3.7 days to the Tijuana border. “Transport type” and “travel companions,” show different directions when compared to “violence.” Particularly for the case of central american women, most of them traveled intermittently by bus and train.

## Discussion

According to the U.S. Custom and Border Protection information, there were 244,332 apprehensions of Central American migrants on the southwestern border of Mexico who were seeking to cross through the country, to different border points with the USA [15]. A report by Doctors Without Borders indicated that the main countries of origin of migrants deported from the USA in 2019 were as follows: first, Mexico (65%) and second, the countries that make up the Northern Triangle of Central America (29%). ), which includes, in order of frequency, Honduras, El Salvador, and Guatemala. This indicates, among other things, the enormous mobility of Mexican nationals to the USA, who joined the transit of Central Americans. This partially coincides with the information collected in the present study, where although Mexicans were the largest study group, for the group of Central Americans there were not so many people from Honduras, but quite a few from Guatemala and Nicaragua [7, 16]. It should be noted that the USA has not been the preferred site for Nicaraguans in recent years, who usually migrate mainly to Costa Rica [4, 8].

According to the Immigration and Customs Enforcement (ICE), the typical deported individual (76%) is a young person, between 20 and 39 years old [17] and predominantly male (84%) [4], generally being women of lower age ranges than men. In our study, it coincides that Central American migrants, especially women, are younger than Mexican ones, who do not appear in the age groups after 48 years old. In addition, we found that the reasons that lead them to travel to the USA are usually related to violence. Mexican women migrate mainly for economic and family reunification reasons, perhaps due to this last aspect, the predominance in relation to men can be understood, after 48 years old, being that most of Mexican migrants (78%), have family networks and/or friends in the United States.

These high rates of predatory violence by the organized crime have a severe impact on migrants health. Along the migratory route they are victims of different types of violence. The most frequent one is physical, such as injuries due to beatings, robberies, kidnappings, and extortion, followed by the psychological one, where there are threats, insults, and humiliations based on stereotypes. The foregoing, without neglecting sexual violence, which includes sexual harassment, rape, and the demand for sexual favors [18–20]. These findings match our results, which report robberies first, followed by physical, psychological, and sexual violence. In all cases, the most affected were Central American women.

According to the UN Human Rights Council in 2016, women in transit have a very high probability of suffering sexual abuse [21]. Amnesty International has even reported that one out of six women suffers sexual violence during this journey through Mexico, which forces Central American women to prepare physically and psychologically so that if they are raped, they can continue their crossing through our country [13]. Our research shows alarming results, since proportionally, it was observed that one in four Central American women were sexually violated (the majority raped), but not so for the Mexican women. In the case of men, rape has been poorly documented [22]; however, in this study a proportion of less than 0.1 of men sexually violated (raped) was found, but only in Central Americans.

Some authors have reported that the travel time throughout the country, from their place of origin, can last between 3 days and 3 months [23, 6]. What coincides with the results of this investigation, but there is a difference according to the nationality, for Mexicans this time was 3.7 days and for Central Americans almost 4 months. According to these results, it seems that traveling with children is a protective factor for migrant women, who shortened their trip by an average of 1 month. The erratic nature of the journey in Central American people and the duration time generates a greater probability of suffering violence in its different types. This factor, together with the type of transportation, had a significant association according to the principal component analysis, for a greater risk of being violated in its different forms, not only sexually.

Access to health systems for the migrant population is a fundamental human right that must be preserved by each government of the countries of transit and destination. One of the objectives of public health is to eradicate the effects of inequality by facilitating access to health services for the entire population. In the case of Mexico, there is a “Comprehensive Health Care Plan for the Migrant Population” which adheres to the international framework of the pacts and treaties signed by Mexico, which are focused on protecting the health of migrants [24], as well as the Beta Protection Groups or the Integration Centers for Migrants (CIM) and the recent Action Plan for the USA-Mexico Bicentennial Framework [25]. However, having these government strategies has not ensured that the migrant population that suffers violence can access health systems. It stands out that in this study only half of the people who suffered violence in its different forms had access to medical care.

Despite the fact that Mexicans represent the group with the highest number of immigrants in the USA, the number of detentions and deportations in them is much lower (38.5%) compared to other nationalities (61.5%) [26]. This proportion represents a contrast with the sample universe of this study, where 88% of the respondents were Mexican and 12% Central American. This difference could be due to the fact that in the Chaparral customs, the interviewer was not allowed to access the area of Central American deportees; he was only allowed to enter and carry out surveys in the area of Mexican deportees although, in the shelters visited, there was no restrictions. This circumstance constituted a limitation of the study observable in the sample, as well as the fact that the COVID-19 pandemic prevented the continuation of the survey due to the quarantine. Another limitation was that the study was only carried out in the city of Tijuana and that the results could vary in other border cities (social, environmental, geographical risks, etc.). The lack of privacy to interview migrants when asking the questions and the fact that the questionnaire did not characterize the type of sexual violence they suffered (sexual harassment, inappropriate touching, gang rape or sexual exploitation, among others) limited the characterization of sexual abuse. However, most people interpreted sexual violence as rape.

Importantly, many of the migrants die in transit through Mexico because of violence [27, 28]. However, the magnitude of the problem cannot be documented, since there are no official records that allow identifying or quantifying the number and characteristics of the dead or disappeared people who entered the country and that by not being able to collect their life stories, the impact of violence on this population becomes incalculable [29].To conclude, this paper documents the greater vulnerability of Central American migrants compared to Mexicans, especially women, who were proportionally the most aggrieved victims in all types of violence and crimes (robbery, kidnapping, sexual, psychological and physical violence). In many cases, women suffered all types of violence during their transit.

## Conclusions

Understanding the violence suffered by migrants during their transit through Mexico is a complicated issue because it has a multifactorial character, where economic, political, and social aspects are mixed, which lead to structural problems that are difficult to solve. However, it is striking that Mexico is one of the countries in Latin America that has the largest number of norms, programs, and policies for the protection of migrants and that in practice do not work for this purpose. In this context, it is urgent to implement new mechanisms for the protection of migrants, as well as for the regulation and access to health services, not only for nationals, but also for foreigners, especially women.

### Aknowledgements

The authors thank the Ministry of Foreign Affairs, responsible for the Chaparral Customs and the shelters: Casa de los Pobres A.C., Casa del Migrante, Salesian breakfast bar &quot, Padre Chava&quot, Instituto Madre Asunta A.C., and Ejército de Salvación A.C., in the City of Tijuana, for the facilities granted for the survey for this research.

1. Yossadara Luna Téllez ^1´^ https://orcid.org/0000-0001-5622-663X

2. Andrea Macías Silva ^2^ https://orcid.org/0000-0003-1571-2839

3. Flor Mariana Sanchez Nuñez ^3´^ https://orcid.org/0000-0002-9035-4675

1´. Universidad Autónoma de Baja California, Tijuana, México.

2´. Instituto Mexicano del Seguro Social, Tijuana, México.

3´. Unidad de Medicina Familiar 18, Instituto Mexicano del Seguro Social, Tijuana, México.

## Data Availability

Data supporting the findings of this study are available from the Center for Research in Politics, Population and Health of the National Autonomous University of Mexico.
